# Supplementary material for: Male autism spectrum disorder is linked to brain aromatase disruption by prenatal BPA in multimodal investigations and 10HDA ameliorates the related mouse phenotype
Source: Nat Commun. 2024 Aug 7;15:6367. doi: 10.1038/s41467-024-48897-8 (PMC11306638; doi:10.1038/s41467-024-48897-8)
Supplement: Supplementary file 1 — Supplementary Information [file 41467_2024_48897_MOESM1_ESM.docx]

Supplementary Information for

**Male autism spectrum disorder is linked to brain aromatase disruption by prenatal BPA in multimodal investigations and 10HDA ameliorates the related mouse phenotype**

**Authors:** Christos Symeonides^1-3^†, Kristina Vacy^4-5^†, Sarah Thomson^4^, Sam Tanner^4^, Hui Kheng Chua^4, 6^, Shilpi Dixit^4^, Toby Mansell^2,7^, Martin O’Hely^2,8^, Boris Novakovic^2,8^, Julie B Herbstman^9,10^, Shuang Wang^9,11^, Jia Guo^9,11^, Jessalynn Chia^4^, Nhi Thao Tran^4^, Sang Eun Hwang^4^, Kara Britt^13-15^, Feng Chen^4^, Tae Hwan Kim^4^, Christopher A Reid^4^, Anthony El-Bitar^4,^ Gabriel B Bernasochi^4,16^, Lea M Durham Delbridge^16^ , Vincent R Harley^13,17^, Yann W Yap^6,17^, Deborah Dewey^18^, Chloe J Love^8, 19^, David Burgner^2,7,20-21^, Mimi LK Tang^2,16^, Peter D Sly^8,22-23^, Richard Saffery^2^, Jochen F Mueller^24^, Nicole Rinehart^25^, Bruce Tonge^26^, Peter Vuillermin^2,8,19^, the BIS Investigator Group, Anne-Louise Ponsonby^2-4^ and Wah Chin Boon^4,27^

These authors contributed equally: Christos Symeonides, Kristina Vacy.

These authors jointly supervised this work: Anne-Louise Ponsonby, Wah Chin Boon.

**Affiliation**

^1^Minderoo Foundation; Perth, Australia.

^2^Murdoch Children’s Research Institute, Parkville, Australia.

^3^Centre for Community Child Health, Royal Children's Hospital; Parkville, Australia

^4^The Florey Institute of Neuroscience and Mental Health; Parkville, Australia.

^5^School of Population and Global Health, The University of Melbourne; Parkville, Australia.

^6^The Hudson Institute of Medical Research; Clayton, Australia.

^7^Department of Pediatrics, The University of Melbourne; Parkville, Australia.

^8^School of Medicine, Deakin University; Geelong, Australia.

^9^Columbia Center for Children’s Environmental Health, Columbia University, New York, NY, United States.

^10^Department of Environmental Health Sciences, Columbia University, New York, NY, United States.

^11^Department of Biostatistics, Columbia University, New York, NY, United States.

^12^The Ritchie Centre, Department of Obstetrics and Gynaecology, School of Clinical Sciences, Monash University; Clayton, Australia.

^13^Department of Anatomy and Developmental Biology, Monash University; Clayton, Australia.

^14^Breast Cancer Risk and Prevention Laboratory, Peter MacCallum Cancer Centre; Melbourne, Australia.

^15^Sir Peter MacCallum Department of Oncology, The University of Melbourne; Melbourne, Australia.

^16^Faculty Medicine, Dentistry & Health Sciences, University of Melbourne; Parkville, Australia.

^17^Sex Development Laboratory, Hudson Institute of Medical Research; Clayton, Australia.

^18^Departments of Paediatrics and Community Health Sciences, The University of Calgary; Calgary, Canada.

^19^Barwon Health; Geelong, Australia.

^20^Department of General Medicine, Royal Children’s Hospital; Parkville, Australia.

^21^Department of Pediatrics, Monash University; Clayton, Australia.

^22^Child Health Research Centre, The University of Queensland; Brisbane, Australia.

^23^WHO Collaborating Centre for Children's Health and Environment; Brisbane, Australia.

^24^Queensland Alliance for Environmental Health Sciences, The University of Queensland, Brisbane Australia

^25^Monash Krongold Clinic, Faculty of Education, Monash University; Clayton, Australia.

^26^Centre for Developmental Psychiatry and Psychology, Monash University; Clayton, Australia.

^27^School of BioSciences, Faculty of Science, The University of Melbourne; Parkville, Australia.

Correspondence to: [wah.chin.boon@florey.edu.au](mailto:wah.chin.boon@florey.edu.au) (lab studies)

[annelouise.ponsonby@florey.edu.au](mailto:annelouise.ponsonby@florey.edu.au) (human cohort studies)

**This PDF file includes:**

Supplementary Materials and Methods

Supplementary Figs. 1-17

Supplementary Tables 1-5

Supplementary References

**Supplementary Materials and Methods**

**HUMAN STUDIES**

**Genome-wide DNA methylation arrays and analysis**

The Illumina Infinium MethylationEPIC BeadChips (referred to from now as ‘EPIC array’) was used for DNA methylation profiling of cord blood from the BIS cohort. Genomic DNA (200 to 500 ng) from cord blood was randomized into 96-well plates and sent to the Kobor laboratory (Canada) for sodium bisulfite treatment and processing on the EPIC array. The EPIC array measures DNA methylation level at more than 850,000 CpG sites (referred to as ‘EPIC probes’), and covers all gene promoters, gene bodies and ENCODE-assigned distal regulatory elements^1^. Raw IDAT files were processed and analyzed using the MissMethyl and minfi packages for R^2, 3^, both available from Bioconductor^4^. Samples were checked for quality and those with a mean detection p-value of >0.01 were removed (128 samples), leaving 946 cord blood samples for analysis. Data were normalized for both within and between array technical variation using SWAN (Subsetquantile Within Array Normalization)^5^. Probes with poor average quality scores (detection *P*-value > 0.01) and cross-reactive probes^1^ were removed from further analysis. This left a total of 798,259 probes for cord blood analysis. Cell composition was determined using the estimateCellCounts tool, with the ‘CordBlood’ reference data used for neonatal blood spot analysis^6^. Maternal contamination of infant cord blood was based on a CpG signature^7^.

Aromatase *CYP19A1* brain promoters PI.f (primary) and PII mapped to flanking positions chr15:51570141-51570603^8^ and chr15:51535243-51535504^9^ respectively in human-genome build GRCh37/hg19. Average methylation around each promoter was assessed using an expanding window, beginning with CpGs located within each promoter sequence and extending to 5, 7, 11 and 15 CpG overlaps, and finally to the whole aromatase promoter region. Average methylation of PI.f and PII jointly was also assessed.

BPA values were corrected for specific gravity and log base 2 transformed, and association with each methylation window was tested in R using multiple linear regression. Each model incorporated the following covariates: sex, year of birth, mother’s gestational age, time of day when blood sample was taken, maternal contamination, cell-type proportions for B cells, CD4 T cells, CD8 T cells, and granulocytes. To control for genetics, the mQTLs rs7181429 (for promoter PI.f) and rs61018102 (for promoter PII), identified using the Human Whole Blood mQTL Atlas^10^, were also added. Inclusion of natural killer cells, monocytes and nucleated red blood cells did not alter any reported association.

Concordance between methylation of aromatase in blood and brain tissue was evaluated using the web-based tool IMAGE-CpG^11^.

**LABORATORY STUDIES**

**SHSY-5Y** **cell culture study**

**Cell treatment**

Cell treatments were performed when cells reached 90% confluence (4.36x106 cells/mL). In each trial, there were three technical replicates of each treatment group.

Media was prepared as per all cell culture experimentation. Five 75 cm^2^ cell culture flasks (353136, BD Falcon, Pennsylvania, USA) were prepared and 75 mL of media was pipetted into each of these flasks. These flasks represented each one of the treatment groups in a set. Subsequently, when the appropriate amount of drug and vehicle control was pipetted into each of the five flasks, 75 mL of media from each flask was divided equally (25 mL each) amongst three T-175 cell culture flasks. Cells were incubated with the chemical for five days (the mouse estrus cycle is 4-5 days)^12^.

**Protein Assay**

Protein concentrations were quantified using the Bio-Rad DC Protein Assay (Catalog No. 500-0111, Bio-Rad, California, USA). Protein samples were prepared by a 1/5 volume dilution in 1x Dulbecco’s Phosphate Buffered Saline (DPBS), constituting 4 μl of the protein sample to 16 μl of 1x DPBS. A 5 μl aliquot of each diluted sample was then allocated to a 96-well microplate with a Nunclon delta surface (Part No. 163320, Thermo Scientific, Massachusetts, USA). Simultaneously, a series of Bovine Gamma Immunoglobulin standards were constituted at concentrations of 2 mg/ml, 1 mg/ml, 0.5 mg/ml, 0.25 mg/ml, 0.125 mg/ml, along with a blank (1xPBS). Subsequently, 5 μl of each standard solution was dispensed into the corresponding wells of the microplate. For the development of the assay, 2450 μl of Reagent A was blended with 50 μl of Reagent S, and 25 μl of the resultant mixture was pipetted into each well. This was followed by the addition of 200 μl of Reagent B to all wells. The microplate was then incubated in darkness for 15 minutes to permit color development. Post-incubation, the absorbance was recorded at a wavelength of 750 nm utilizing the Benchmark Plus microplate spectrophotometer (Bio-Rad, California, USA). Following each assay, a standard curve was generated to calibrate the absorbance readings against protein concentration.

Protein samples were then prepared for Sodium Dodecyl Sulfate-Polyacrylamide Gel Electrophoresis (SDS-PAGE). Protein samples encompassed 15 distinct identities per set, equilibrated to 4°C prior to processing. For assay preparation, each sample was constituted to a final volume of 30 μl. This volume included 3 μl of 2-Mercaptoethanol (Cat No. M3148-100ML, Sigma Aldrich, St Louis, MO, USA) for reduction, 4 μl of an 8x concentrated loading dye for visual tracking, and an aliquot of protein sample as determined by prior quantification, with the balance comprised of DPBS. Following the addition of 2-Mercaptoethanol, samples were subjected to thermal denaturation at 100°C for 5 minutes on a heating block. Post-denaturation, samples were centrifuged using an Eppendorf Centrax centrifuge to consolidate the contents.

**Sodium Dodecyl Sulfate-Polyacrylamide Gel Electrophoresis (SDS-PAGE)**

SDS-PAGE was used to segregate proteins based on molecular mass under denaturing conditions. Assembly of the gel apparatus commenced with the securing of a short and spacer plate, spaced 1.5mm apart, within a gel casting frame, confirming water-tight seals via a distilled water trial. A dual-layer gel system was constructed, with a lower percentage stacking gel atop a higher percentage resolving gel. For the resolving gel (10% acrylamide), the polymerization was initiated by the addition of ammonium persulfate (APS) and N,N,N',N'-tetramethylethylenediamine (TEMED) catalyzation. Upon thorough mixing, it was cast between the plates, ceasing 1 cm below the plate's crest and overlaid with distilled water to prevent oxygen inhibition. Incubation ensued for 20 minutes at 37°C for gel polymerization.

Subsequently, APS and TEMED were admixed with the stacking gel (4% acrylamide) precursor solution, promptly pipetted above the resolved layer and crowned by the insertion of Teflon combs, carefully expelling entrapped air. Gel solidification was achieved at ambient conditions over 20 minutes.

Following comb removal, the gel cassette was anchored into the electrophoresis chamber, nestled within the tank. Buffer chambers were then suffused with 1x running buffer, concocted from 1800 ml distilled water and 200 ml of a 10x stock (comprised of 60 g TRIS, 296 g glycine, and 20 g SDS in 2 L of distilled water).

Electrophoretic separation was conducted by loading 1 μl of protein ladder into the inaugural well, succeeded by the introduction of 30 μl of each protein specimen into subsequent wells, following a predefined sequence of untreated (UT), osmotic (O), ½-fold FDA, full-dose FDA, and double-dose FDA. Samples were dispensed using specialized protein/electrophoresis tips (Cat no. 223-9915, Bio-Rad, California, USA).

The gel was subjected to a two-phase electrophoretic migration, initially at 60V through the stacking gel, followed by 100-120 minutes at 100V through the resolving gel. Power was supplied by a PowerPac High Current power supply (Bio-Rad, Catalog 164-5052), and separation was carried out using a Mini-PROTEAN Tetra Cell (Catalog no. 165-8006, Bio-Rad, California, USA).

For the resolving gel, a 10% solution was prepared, optimized for the resolution of proteins ranging from 16,000 to 70,000 Daltons. To compose a single 1.5 mm gel, the following components were combined: 3.3 ml of 30% acrylamide mix, 4.03 ml of water, 2.5 ml of 1.5M TRIS-HCl (pH 8.8), 0.1 ml of 10% SDS, 0.05 ml of 10% ammonium persulfate (APS), and 0.02 ml of TEMED. A 4% stacking gel was used to concentrate proteins before their entry into the resolving gel. The stacking gel mixture for a single 1.5 mm gel was constituted of 0.5 ml of 30% acrylamide mix, 3.18 ml of water, 1.26 ml of 0.5M TRIS-HCl (pH 6.8), 0.05 ml of 10% SDS, 0.025 ml of 10% APS, and 0.01 ml of TEMED.

**Western Blotting**

Following the SDS-PAGE, proteins were transferred from the gel to a PVDF membrane. The transfer process commenced by filling a container halfway with transfer buffer, a mixture of 1400 ml distilled water, 400 ml methanol, and 200 ml of a 10x transfer buffer solution containing 60g TRIS and 296g glycine dissolved in 2L of distilled water. Filter papers, cut to the size of the gel, were pre-wetted in the 1x transfer buffer. Concurrently, a precut nitrocellulose membrane was activated in 100% methanol before equilibration in the same transfer buffer.

Upon disassembling the electrode assembly and unclamping the plates, the gel was carefully maintained on the spacer plate. A transfer cassette was then assembled in the following order, starting from the top exposed to air: the clear side of the cassette, a sponge, a piece of the pre-wetted filter paper, the membrane, the gel, another filter paper, a second sponge, and finally, the black side of the cassette submerged in the transfer buffer. Ensuring no air bubbles were trapped, the cassette was clamped shut while submerged.

The cassette was then placed into the transfer assembly, aligning black sides together, and the tank was filled with the transfer buffer. To maintain a low temperature and facilitate efficient transfer, an ice pack and a magnetic stirrer were added to the tank. The transfer proceeded at 100V for 1 hour with constant stirring.

Post-transfer, the membrane was carefully extracted from the cassette and trimmed to fit a vectashield tray, ensuring it remained moist by rinsing it in 1x TBST. The membrane underwent a blocking step in a solution comprising 25g of 5% skim milk, 500ml of 1x TBST, and 1ml of 10% NaN3 for 30 minutes on a rocking platform at room temperature. Subsequent to blocking, the membrane was washed thrice with 1x TBST for 10 minutes each to remove unbound blocking agent.

For the antibody probing, the membrane was incubated overnight at 4°C with primary antibodies in a specified dilution. The primary antibodies used were an anti-Aromatase antibody produced in rabbit (1:2000; cat# A7981; Sigma Aldrich, St. Louis, MO, USA) and an anti-β-Actin antibody produced in mouse (1:10,000; cat# A5316; Sigma Aldrich, St. Louis, MO, USA). Each antibody was diluted in a solution containing 10ml of 1x TBST and 0.30g of Albumin from bovine serum (cat# A3912-100G , Sigma Aldrich, St. Louis, MO, USA). Following overnight incubation, the blots were washed and subsequently incubated with the corresponding secondary antibodies: IRDye 800CW Goat anti-Rabbit IgG (cat# 926-32211; LI-COR Biosciences, Lincoln, NE, USA) for anti-Aromatase and IRDye 680RD Goat anti-Mouse IgG (cat# 926-68070; LI-COR, Biosciences, Lincoln, NE, USA) for anti-β-Actin. Post-secondary antibody incubation, the blots were washed again in 1x TBST to remove unbound antibodies, preparing them for detection.

The membranes were washed with 1X TBST prior to imaging on an Odyssey infrared imaging system (LI-COR Biosciences, Lincoln, NE, USA). Fluorescence quantification was performed with the Image Studio™ Lite software. Quantitative assessments were standardized against the housekeeping protein β-actin.

**Animal studies**

**Behavioral paradigms**

**Y-Maze**

Following a three-day acclimation period following 3-chamber testing, a subset of mice underwent the Y maze assessment. The purpose of this test was to test whether differences in sociability could be explained by a short-term spatial memory deficit. BPA exposed pups were aged P27 to P30, and ArKO mice treated with estrogen or sham pallet were aged PND34-36 at the time of testing. Both male and female mice were tested. This test involves an apparatus with three arms, each designated differently as the home arm, familiar arm, and novel arm. To mitigate potential side bias in the apparatus, the designation of the arms was randomized between mice. The assessment comprised two trials, each lasting 10 minutes. Mice were placed in the home arm at the start of each trial. In trial 1, the mouse could freely explore the home and familiar arm, and the novel arm was made inaccessible. There was an inter-trial period of 10 minutes where the mouse was returned to home cage. During trial 2, the mouse could access all three arms. The body center point was tracked and quantified by TopScan Lite (Clever Sys Inc., Reston VA, USA). In this study, the key measure extracted was the duration of the body in each arm. Time spent in the novel arm was compared to the familiar arm for each mouse to confirm more time was spent in the novel arm. Then the percentage of time spent in the novel arm was compared between groups.

**Water squirt induced grooming**

Mice were acclimatized to a clean, empty clear IVC mouse cage bottom (Techniplast GM500 Mouse IVC Green Line) for 10 minutes. Both male and female mice were tested. Then mice were lightly misted with room temperature water twice using a spray bottle aiming at the top center of the mouse’s body. Video footage was recorded for the following 10 minutes (side on view) for an observer blind to treatment to later measure the duration and bouts of grooming using a stopwatch.

**Visualizing c-Fos activation to conspecific exposure (amygdala)**

**Histology**

Mice were deeply anesthetized with the inhalant anesthetic isoflurane (IsoFlo^TM^, Abbott Laboratories, Victoria, Australia) and perfused 2 hours after stranger/novel object exposure was complete. Once foot reflexes were absent, the animal was transcardially perfused with 15 mL phosphate-buffered saline (PBS) followed by 40 mL of 4% paraformaldehyde (PFA) in PBS containing 0.2% picric acid as the fixative. After perfusion, brains were rapidly removed from the skull and placed in 10 mL of 4% PFA solution for approximately 4 hours on ice. Brains were then transferred into 20% sucrose solution for 3 days to dehydrate prior to freezing. Brains were then frozen in a dish of isopentane (Fisher Scientific, MA, USA) cooled on dry ice, wrapped in foil, and stored at -80°C. Prior to sectioning, brains were kept at -20°C overnight. A notch was made in the cortex of the left hemisphere. A Leica CM1850 (Wetzlar, Germany) cryostat was used to section the brain into 40$\mu$m coronal sections, bregma -0.7 to bregma -2.8. Sections were collected as 4 series into a 24 well Nunc microwell plate (Nuncon Delta, Denmark) containing cryoprotectant (30% ethylene glycol, 30% glycerol, .05M PBS). The sections were stored at -20°C until they were immunostained and mounted.

*Cyp19*-EGFP brain sections were double-stained with primary antisera EGFP and c-Fos. Sections were rinsed (2×5 min) in 0.01M PBS followed by Triton-X (1x5 min) and incubated for 96 hours at 4°C under gentle agitation with chicken anti-EGFP antibody (1:1,000; cat# ab13970; Abcam, United Kingdom) and rabbit anti-c-Fos (1:2000; cat# ABE457; Calbiochem, USA). Antibodies were diluted in 0.01M PBS containing 1.5% Triton X-100 and 0.5% donkey serum. Sections were then rinsed in 0.01M PBS (2x5 min) followed by blocking with 6% donkey serum and 3% Triton X in 0.01M PBS (1x10 mins) and incubated for 4 hours at room temperature under gentle agitation with secondary antibodies: donkey anti-chicken Alexa488 (1:200; cat# A78948, Invitrogen, USA) and donkey anti-rabbit Alexa594 (1:200; cat# A-21207; Invitrogen, USA). Sections were rinsed in 0.01M PBS (2x5 min), mounted, and coverslipped using a fluorescent mounting medium (Dako, Glostrup, Denmark).

ArKO sections were immunostained for c-Fos according to the protocol described above for *Cyp19*-EGFP mice, except chicken anti-EGFP antibody was not added, nor was anti-chicken Alexa488.

After immunostaining, the posteriodorsal medial amygdala (PD MeA) was visualized under a microscope. Photographic images of the PD MeA were taken for all *Cyp19*-EGFP mice sections. ArKO mice sections were analyzed with stereology to estimate the number of c-Fos positive cells in the PD MeA.

**Stereological analysis**

Stereology was done on a Leica DMLB2 fluorescence microscope (Ludl Electronics, Hawthorne, NY, USA) and HV-C20AMP color video camera (Hitachi, Tokyo, Japan). The program used for stereological analysis was StereoInvestigatorTM 8.5 (MBF Bioscience, Williston, VT, USA).

Each section containing the medial amygdala was delineated with the guidance of Paxinos and Franklin Mouse Brain Atlas^13^ and stereological analysis were performed to estimate the total c-Fos positive number for the medial amygdala. C-Fos positive cell numbers were estimated using the Fractionator method on StereoInvestigatorTM 8.5. Immunostained, c-Fos cells were identified as those with a large clear cytoplasm around 20 μm in diameter and prominent nucleoli contained within a pale nucleus (neuron) with red fluorescence (c-Fos positive). The experimenters were blind to experimental group during stereological analysis.

**Cell (neuron) counting**

**Brain collection**

BPA exposed (ED10.5-14.5) *Cyp19*-EGFP and untreated ArKO mice, not used in behavioural experiments, were sacrificed between PND27-31. Once anesthetized by isofluorane (IsoFlo^TM^, Abbott Laboratories, Victoria, Australia) inhalation, animals were perfused transcardially with approximately 15 mL of phosphate buffer (PB: 19.0 mM NaH_2_PO_4_.H_2_O, 80.7 mM Na_2_HPO_4_) solution, followed by about 40 mL of 4% paraformaldehyde (PFA). Brains were dissected out and placed in 4% PFA solution for 4 hours at 4°C, then transferred into 20% sucrose solution (in dH_2_O) to dehydrate.

**Tissue processing and staining**

After dehydration in 20% sucrose, perfused brains were drop frozen in a dish of isopentane (Fisher Scientific, Waltham, MA, USA) cooled in dry ice, then wrapped in foil for storage at -80°C. At random, one male and female brain was chosen from different litters and thawed to -20°C overnight. Brains were notched in the cortex of the left hemisphere and coronal 40 μm sections were cut rostrocaudally at -21°C using a Leica CM1850 cryostat (Wetzlar, Germany) with a 1 in 4 series collection into cryoprotectant containing (30% ethylene glycol, 30% glycerol, 0.05M PB) 24 microwell plates (Nunc ®, Nuncon Delta, Denmark) beginning approximately at bregma -0.70 until -2.80. Sectioned brains were stored at -20°C.

**Cresyl Violet staining**

Through systematic uniform sampling every 4^th^ section was selected and mounted in bregma order onto gelatin slides (Menzel-Gläser, Germany) and left to set at room temperature overnight. All sections were then stained with Cresyl Violet (GURR/BDH Ltd., Poole, BH, UK) in one lot to ensure uniformity of the staining intensity across all specimens, and then coverslipped.

**Brain region identification and delineation**

As the cytoarchitecture of the mouse posterior medial amygdala had not yet been characterized in the literature, it was paramount to establish reliable criteria prior to analyzing test mice brains. Practice brain sections from P30 WT mice were used to establish consistency in identifying and delineating not only the posteriodorsal medial amygdala (PD MeA), the nucleus of interest, but also the characteristics of surrounding hallmark structures at each bregma such as the hippocampus, third and lateral ventricles and the basolateral amygdaloid nucleus. Once PD MeA boundaries and corresponding bregma levels had been established, the PD MeA could then be reliably and consistently identified in test adult mouse brain sections, with the guidance of Mouse Brain Atlas^13^. Sections were viewed and the left PD MeA delineated using a Leica DMLB2 fluorescence microscope (Leica, Wetzlar, Germany) under brightfield with a x5 objective (N.A. 0.12), due to time constraints, the right PD MeA was not analyzed. Identification and delineation of the left PD MeA throughout the various sections for each brain was conducted blind to experimental group until regions for all brain specimens had been analyzed.

**Stereological analysis**

Every section containing the PD MeA was delineated and stereological analysis performed to assess the volume and estimate the total cell number per brain. The computerized stereology workstation used comprised of a Leica DMLB2 fluorescence microscope, objective lenses x5 (N.A. 0.12), x10 (N.A. 0.45), x20 (N.A. 0.75) and x100 (N.A. 1.30), a motorized specimen stage (Ludl Electronics, Hawthorne, NY, USA), HVC20AMP color video camera (Hitachi, Tokyo, Japan) and StereoInvestigator 8.5 (MBF Bioscience, Williston, VT, USA). Cell numbers were estimated using the Fractionator method^14^. Cells were differentiated and counted as two types; those with a large clear cytoplasm around 20 μm in diameter, prominent nucleoli (commonly 2-6) contained within a pale nucleus were classed as neuronal cells, while those which were around 10 μm with an intensely stained nucleus lacking nucleoli were identified as non-neuronal/glial cells. All cells that came into focus within each of the sampling sites distributed systematically throughout the delineated PD MeA, were counted and labelled as either (1) cells classed as neurons or (2) cells classed as non-neurons/glial cells. StereoInvestigator then calculated an estimate for the total number of neurons, non-neurons, and combined cell number. MeA volume was estimated using Cavalieri’s principle^14^ where delineated PD MeA area in each section was determined, summated and multiplied with the section interval of 4 and the section thickness, 40 μm. The experimenters were blind to experimental group during stereological analysis.


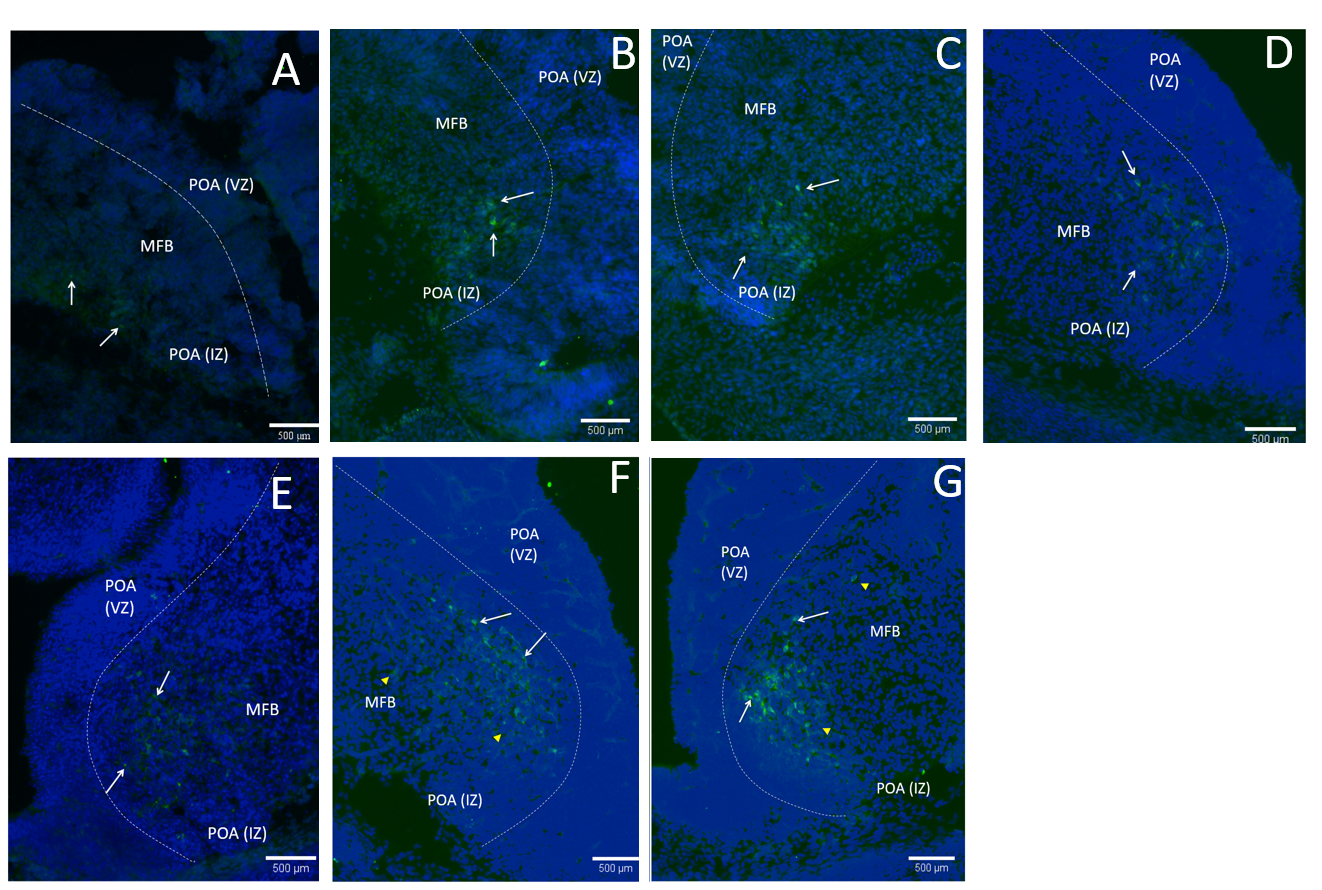


**Supplementary Fig. 1 | Detection of EGFP transgene that identifies aromatase-expressing cells in the embryonic mouse brain.** We detected EGFP as a transgenic marker for aromatase expression in the male fetal brains as early as embryonic day 11.5 (e11.5), as shown. (**A**) *Cyp19*-EGFP and DAPI merge 20X image showing expression of *Cyp19*-EGFP in the MFB (Medial Forebrain Bundle), close to the POA (IZ)(Preoptic Area) (white arrows). (**B**, **C**) *Cyp19*-EGFP and DAPI merge 20X image showing *Cyp19*-EGFP expression in the POA (IZ) (white arrows) of male e11.5 brain section. (**D**, **E**) *Cyp19*-EGFP and DAPI merge 20X image showing expression of *Cyp19*-EGFP in the MFB (white arrows) of the female e13.5 brain section. (**F**, **G**) *Cyp19*-EGFP and DAPI merge 20X image showing strong *Cyp19*-EGFP expression in the POA (IZ) (white arrows) and MFB (yellow arrowheads) of male e13.5 brain. Scale bar is 500 µm.

**Supplementary Fig. 2 | The presence of an EGFP transgene in *Cyp19*-EGFP mice does not affect social interaction.** Mice were tested at P24-P27 in a social approach trial (in the modified 3-Chamber Social Approach Task). There were no significant interactions between the treatment group and EGFP genotype *F* (1, 35) = 0.79, *P* = 0.38 (two-way ANOVA) nor were there differences between genotypes within each treatment group. Data represents mean + SEM. Source data are provided as a Source Data file.

**Supplementary Fig. 3 | BPA exposure during early and late gestation does not alter social approach.** Pregnant dams were exposed to either BPA (dissolved in ethanol and diluted in peanut oil) or vehicle (ethanol in peanut oil) between E10.5-E14.5 daily. (**A**) Dams (*n* = 5 per treatment group) were treated early gestation between E0.5-E9.5. There were no differences in the time spent investigating the stranger mouse between male BPA-exposed (*n* = 25) and vehicle (*n* = 17) offspring and nor between female BPA-exposed (*n* = 9) and vehicle female (*n* = 14). Sex-by-treatment interaction *F* (1,56) = 0.17, *P* = 0.68 (two-way ANOVA). (**B**) Mice were treated late gestation between E15.5-E20.5. No differences between male BPA-exposed (*n* = 19) and male vehicle (*n* = 15) mice, nor were there differences between female BPA-exposed (*n* = 18) and female vehicle (*n* = 15) mice. Sex-by-treatment interaction *F* (1,62) = 0.51, *P* = 0.47 (two-way ANOVA). Data represents mean ±SEM. Source data are provided as a Source Data file.

**Supplementary Fig. 4 | The effects of oral BPA administration.** Dams (*n* = 6 per treatment) were fed with raspberry flavored agar with or without BPA between E10.5-E14.5 daily. Male BPA-exposed mice (*n* = 15) spent less time investigating the sex-matched and age-matched novel stranger mouse as compared with male vehicle controls (*n* = 24, *P* = 0.05 (two-sided Mann Whitney *U* test). No differences were observed between female BPA-exposed mice (*n* = 23) and female vehicle controls (*n* = 21) for the time spent investigating sex-matched and aged-matched novel stranger. Data are mean ± SEM. Source data are provided as a Source Data file.

**Supplementary Fig. 5 | BPA-exposed mice and aromatase knockout (ArKO) mice have male specific deficits in sociability, and the ArKO deficit is reversed by early postnatal estrogen replacement.**

(**A**) There was a trend for female BPA-exposed mice (*n* = 18) to spend more time interacting with the stranger compared to controls (*n* = 25) , *P* = 0.07 Holm-Sidak, but both female groups showed a preference for the stranger compared to the empty cage. (**B**) 17β-Estradiol (E2) treatment was able to ameliorate the male ArKO (*n* = 6) sociability deficit and increased the social approach to WT levels (*P* = 0.03, Holm-Sidak test) but E2 treatment had no effects on WT (*n* = 9). (**C**) There were no differences in sociability or social approach in female ArKO mice (WT *n* = 10, KO *n* = 9) but early postnatal treatment with estradiol (WT *n* = 8, KO *n* = 7) induced a sociability deficit in the female. Data are mean ± SEM. Source data are provided as a Source Data file. Note: Str. = Stranger; E = Empty; Veh = Vehicle.

**Supplementary Fig. 6 | Y-maze performance with 10 minute intertrial period is not altered in BPA exposed or ArKO mice.**(**A**) There were no effects of treatment or sex in the percentage of time spent at the novel arm between male BPA-exposed (*n* = 15) and male vehicle (*n* = 12) mice, or female BPA-exposed (*n* = 17) and female vehicle (*n* = 13) mice. Sex x treatment interaction *F* (1,51) = 1.77, *P* = 0.19 (two-way ANOVA). (**B**) There were no effects of genotype or sex in the percentage of time spent at the novel arm between male ArKO (*n* = 11) and male WT (*n* = 7) mice, or female ArKO (*n* = 10) and female WT (*n* = 8) mice. Sex x genotype interaction *F* (1,32) = 0.50, *P* = 0.49 (two-way ANOVA). Data are mean ± SEM. Source data are provided as a Source Data file.

**Supplementary Fig. 7 | NeuN staining of the medial amygdala.** (**A**) There were fewer neurons in the MeA of male BPA-exposed mice (*n* = 3) compared to WT controls (*n* = 3), *P* = 0.046 (two-sided *t*-test) and (**B**) no statistical significance between male ArKO (*n* = 3) and WT (*n* = 3) littermates, *P* = 0.19 (two-sided *t*-test). Data are mean ± SEM. Source data are provided as a Source Data file.


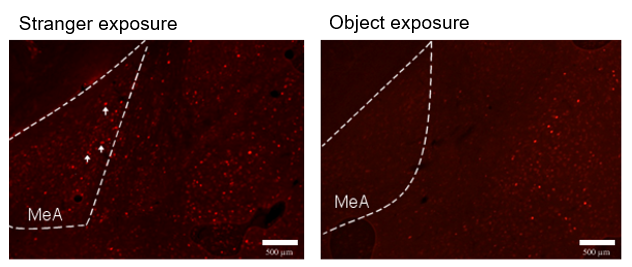


**Supplementary Fig. 8 | Stranger and object exposure in representative slides at 20x magnification.** Representative fluorescence photomicrographs showing c-Fos-Immunoreactivity (red signals, representative signals indicated by white arrows) in P24 untreated male *Cyp19*-EGFP mice exposed to a novel stranger or a novel object (a syringe) in the posteriodorsal medial amygdala (PD MeA). Stranger exposure resulted in c-Fos expression in the MeA whereas object exposure did not induce c-Fos expression. This implies that MeA is activated only after stranger exposure but not object exposure. Scale bar is 500 µm**.**


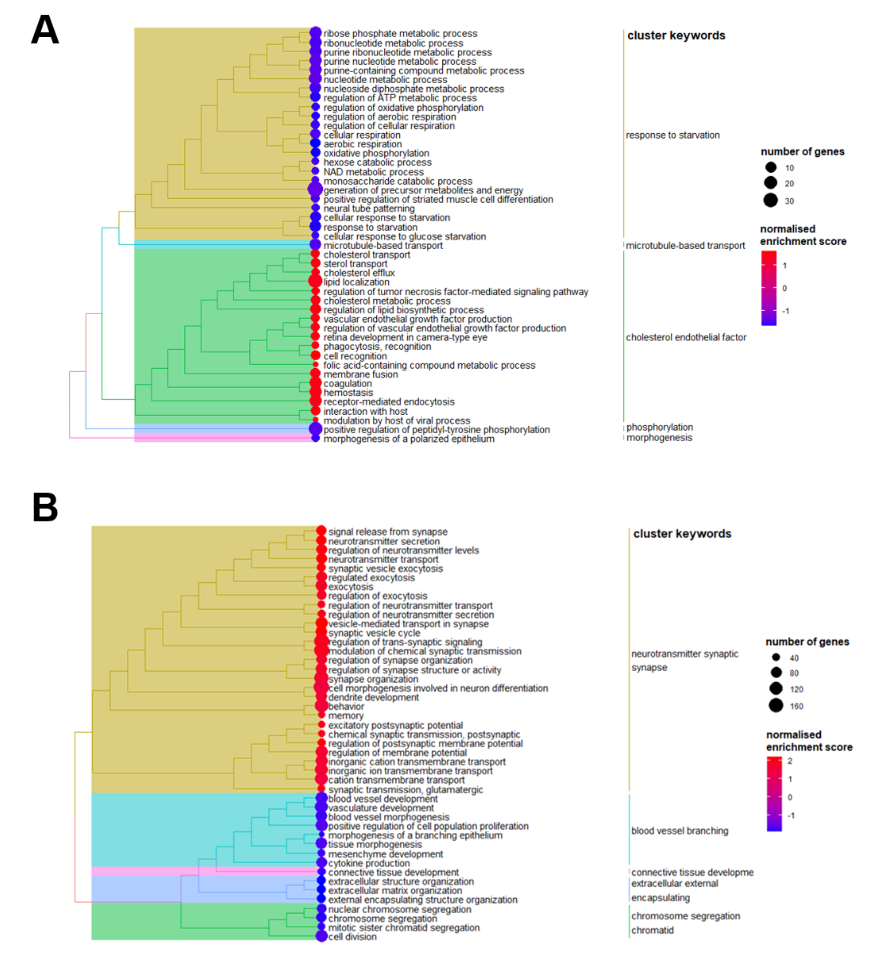


**Supplementary Fig. 9 | Shared brain-related pathways for 10HDA and BPA RNA-sequencing results.**

(**A**) **Gene Set Enrichment Analysis of RNA-sequencing data** reveals BPA induces downregulation of processes related to intracellular respiration and metabolism compared to controls. Brain development has high energy demands, generating a need for optimal metabolic regulation^15^, dysregulation of which has been associated with autism^16^. These findings do not persist after FDR adjustment.

(**B**) **Gene Set Enrichment Analysis of RNA sequencing data** reveals 10HDA induces upregulation of brain, synaptic, and dendritic pathways compared to controls. Of these upregulated mechanisms, 413 persist after FDR correction. Using a candidate pathway approach, we observed that BPA downregulated, and 10HDA upregulated pathways with the keyword ‘axon’ (*n* = 34) or ‘dendrite’ (*n* = 55) more than expected on chance (Fisher’s exact test, *P* = 0.0144 and *P* = 0.0001, respectively).

**Biological processes in ASD pluripotent stem cell model.** We then examined the top mid-gestation biological processes identified to be over-represented in autism compared to non-cases in a previous human pluripotent stem cell analysis^17^. Among the 11 (of 15) pathways also available in our analysis for both BPA and 10HDA, 82% (*n* = 9) were altered in opposite directions for BPA and 10HDA, more than expected by chance (binomial test, *P* = 0.033).


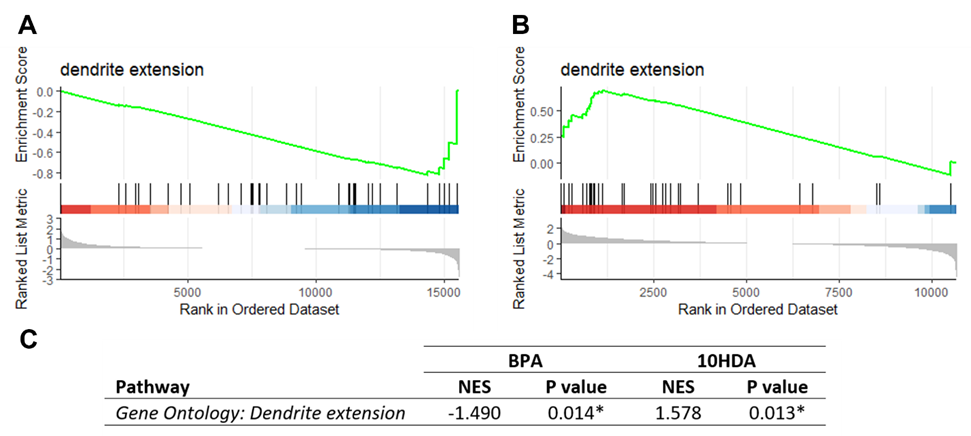


**Supplementary Fig. 10 | Gene Set Enrichment Analysis (GSEA) of RNA-sequencing data.** GSEA of RNA-sequencing data revealed down-regulation of the Gene Ontology ‘dendrite extension’ pathway under BPA but up-regulation under 10HDA exposure. GSEA works by ranking input genes by fold change against a condition of interest and then testing whether genes from a query pathway show significant enrichment at the top (reflected in a positive normalized enrichment score, NES) or the bottom (reflected in a negative NES) of this ranked list. (**A**, **B**) Enrichment-score plots for Gene Ontology ‘dendrite extension’ under BPA and 10HDA exposure, respectively. In each plot, the *x*-axis shows ranking of genes by fold charge, from positive (red) to negative (blue). Genes from the ‘dendrite extension’ pathway (black notches) show a non-random and contrasting distribution, with these genes distributed towards the bottom of the BPA-ranked list, indicating reduced activity, but towards the top of the 10HDA-ranked list, indicating increased activity. (**C**) Enrichment statistics for the ‘dendrite extension’ pathway under BPA and 10HDA exposure, respectively (corresponding to **A** and **B**). BPA and 10HDA were assessed *in vivo* and *in vitro* cell culture, respectively.


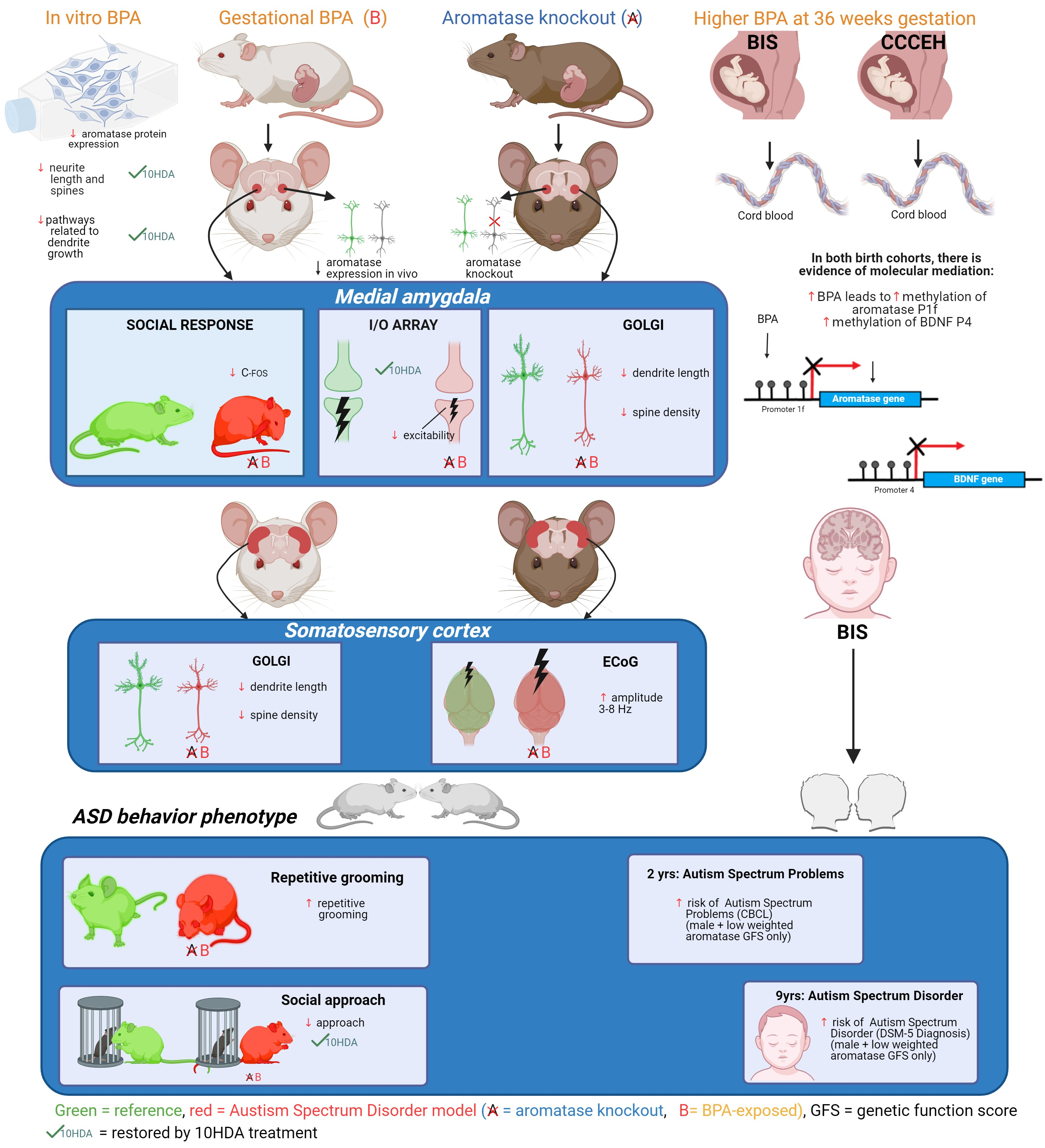


**Supplementary Fig. 11| Summary of key findings for BPA exposure and ASD risk assessment across these studies.** Created with BioRender.com

|  | **BPA-exposed or ArKO**  **Mouse** | **Autism Spectrum Disorder**  **Human** |
| --- | --- | --- |
| **Brain aromatase expression** | **↓** EGFP, marker for aromatase expression in CYP19-EGFP mice (Fig. 3C) | **↓** Brain expression in post mortem ASD brain^18^  **↑** Methylation of brain PI.f promoter in aromatase (associated with **↓** transcription) (Fig. 2) |
| **Amygdala structure** | **↓** Dendritic length (Fig. 5A)  **↓** Amygdala neuron number (BPA exposed, Fig S7) | **↓** Amygdala cell number^19^  **↓** Amygdala cell number in adults^20^ |
| **Amygdala hypo- responsiveness to social stimuli** | **↓** c-Fos (Fig. 5C)  **↓** Multiple electrode analysis  (Fig. 5D) | **↓** fMRI^21^ |
| **Cortical layers** | Excessive neurons in layer 4/5 in somatosensory cortex^8^ | Excessive neurons in layer 4/5 in dorsolateral prefrontal cortex^22^ |
| **Electrophysiological activity** | **↑** ECoG power  At 3-7Hz (Fig. 6D) | **↑** EEG power at 3-9Hz^23^ |
| **Social behavior** | **↓** Social approach: 3 chamber study (Fig. 2A and 2B) | **↑** ASD problems at 2yrs in males with low aromatase GFS (Fig. 1; Table S3)  **↑** ASD diagnosis at 9yrs in males with low aromatase GFS (Fig. 1; Table S3) |

**Supplementary Fig. 12 | Consistency of findings across ASD mouse models in this study and human ASD.** The multi-modal analyses demonstrate similar outcomes in the gestational BPA exposed male mice, male ArKO mice and people with ASD. Note. BPA = bisphenol A; ArKO = aromatase knockout; ASD = Autism Spectrum Disorder, EGFP = enhanced green fluorescent protein; c-Fos = neuronal activation marker; fMRI = functional magnetic resonance imaging; ECoG = electrocorticography; EEG = electroencephalography; GFS = genetic function score of the aromatase enzyme activity. Schematic created with Biorender.com


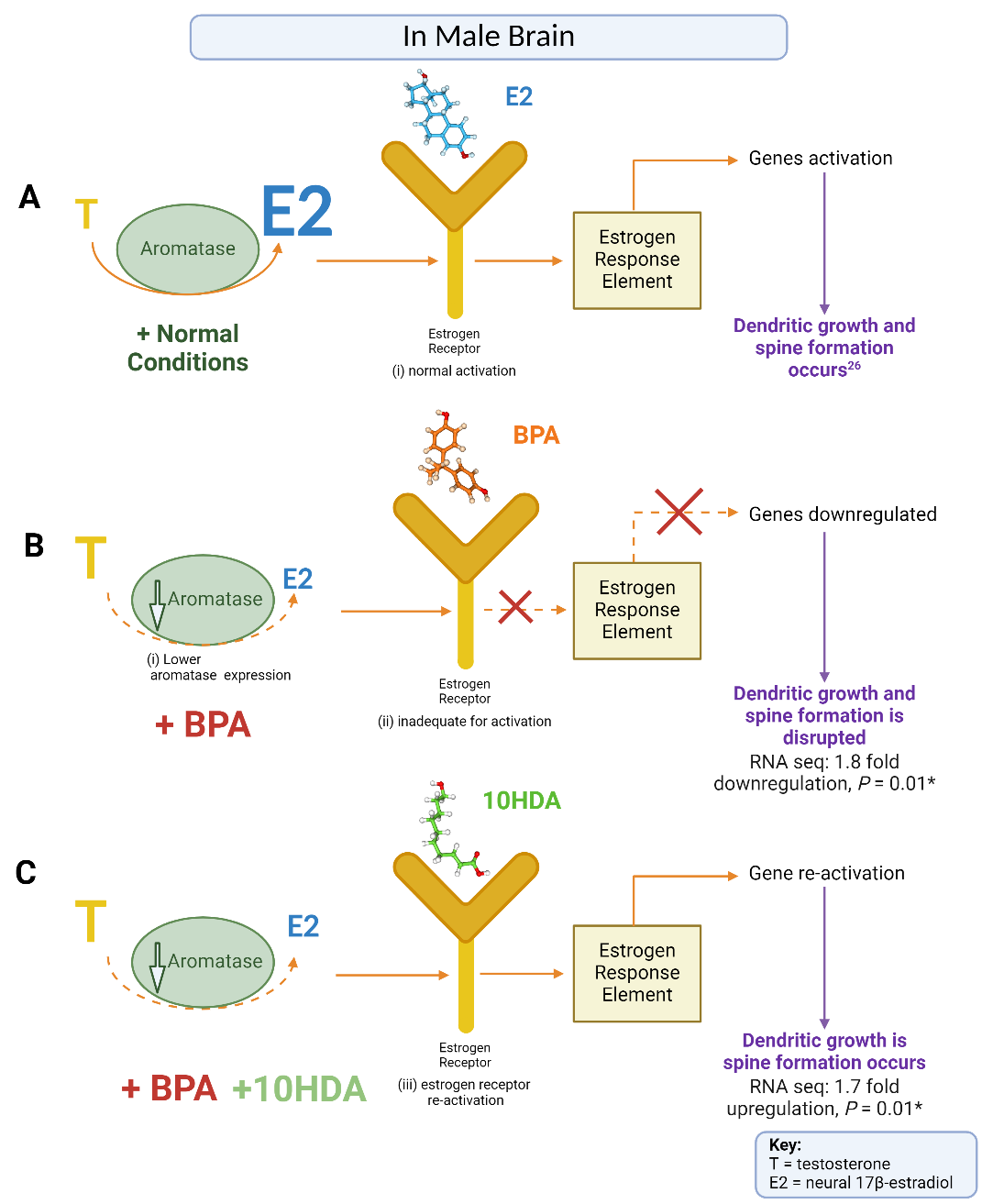


**Supplementary Fig. 13 | A model for the molecular pathway underlying the adverse effect of BPA on dendrite shortening and the opposing effect of 10HDA.** (**A**) Under normal conditions, the brain aromatase converts androgens (e.g., testosterone) to estrogens (e.g., 17β-estradiol) which binds to brain estrogen receptors and trigger a cascade of gene activation via estrogen response elements. The downstream effects include, but not limiting, dendritic growth and spine formation for normal brain functions^26^. (**B**) *In utero* BPA exposure lowers aromatase expression, consequently less estrogen production leading to disruption of dendritic growth and spin formation. BPA itself is sub-optimally estrogenic - less than 1,000-fold compared to estradiol^27^. (**C**) Administration of 10HDA can alleviate the BPA adverse effects by binding to the estrogen receptor and restores normal brain functions such as dendritic growth and spine formation. E2= 17β-estradiol; T =testosterone; BPA =Bisphenol A; 10HDA= 10-hydroxy-2-decenoic acid. Created with Biorender.com

**Supplementary Fig. 14 | Litter sizes by sex.** There were no significant differences between the 0 BPA and 50 BPA litters in the number of males, *P* = 0.51 or females, *P* = 0.98(holm-sidak test). Sex-by-treatment interaction *F* (1,20) = 0.71, p = 0.41 (two-way ANOVA). Data are mean ± SEM. Source data are provided as a Source Data file.

**
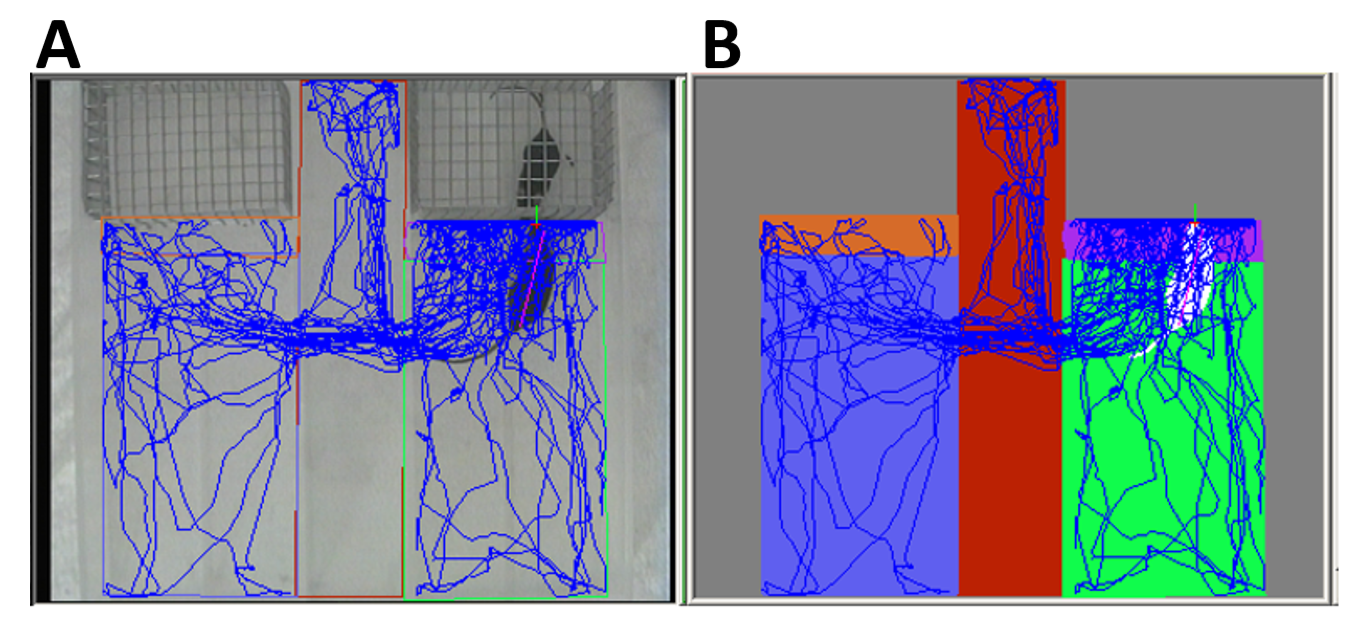
**

**Supplementary Fig. 15. The three chamber social interaction test apparatus.** (**A**) An example of the tracking output, in blue, over the 10-minute trial. The CleverSys^TM^ software tracks the nose point of the mouse. We can observe that the nose point has been tracked more in the stranger’s interaction zone, compared to the empty cage interaction zone. (**B**) The zone overlay in Topscan.


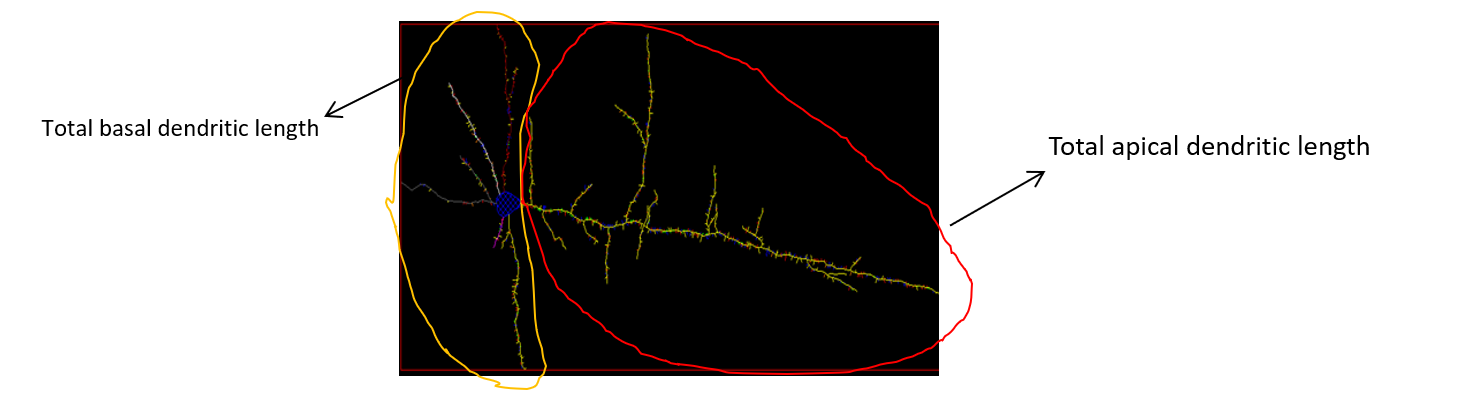


**Supplementary Fig. 16.| Delineation of the basal and apical dendrite measurements.**

**
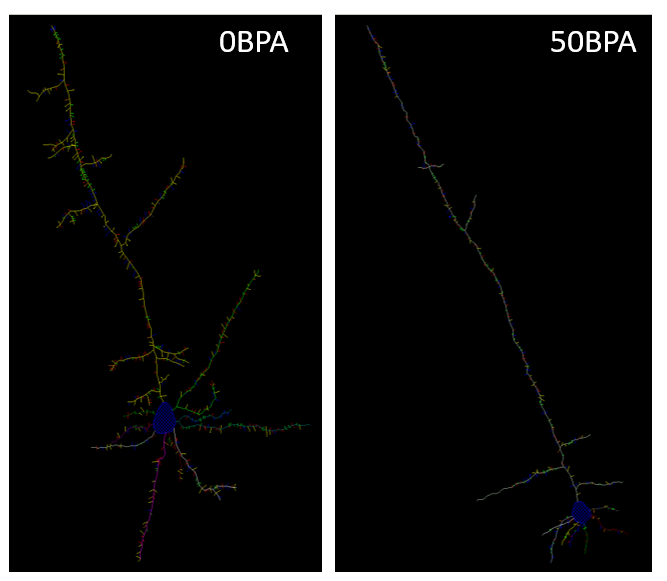
**

**Supplementary Fig. 17.| Representative neural tracing images of the vehicle and BPA treated conditions in the somatosensory cortex.**

| **Characteristic** | **N** | **Mean (SD) or % [n]**  **or *GM (GSD)*** | |
| --- | --- | --- | --- |
| Sex: male^1^ | 1074 | 51.7 | [555] |
| Prenatal BPA in top quartile | 847 | 24.9 | [211] |
| Prenatal urine sample collected after 2pm | 846 | 10.8 | [91] |
| Above median CBCL autistic spectrum problems at 2 years^2^ | 676 | 36.8 | [249] |
| Pediatric DSM-5 ASD diagnosis prior to 9-year phone review^3^ | 827 | 5.20 | [43] |
| Age at BPA measurement (gestational weeks) | 847 | 36.3 | (0.71) |
| Age at CBCL assessment (years) | 676 | 2.46 | (0.15) |
| Age at 9-year phone review (years) | 827 | 9.05 | (0.74) |
| ***Prenatal factors*** |  |  |  |
| Annual household income under $100K AUD | 1041 | 55.4 | [577] |
| Mother’s age at conception (years) | 1074 | 31.3 | (4.79) |
| Mother is university-educated | 1068 | 51.3 | [548] |
| Father is university-educated | 1044 | 35.2 | [367] |
| All grandparents are Caucasian | 1060 | 73.0 | [774] |
| Maternal smoking (persistent throughout pregnancy) | 1061 | 6.31 | [67] |
| Maternal alcohol use (any) | 989 | 52.7 | [521] |
| DEHP exposure ($\mu$g/kg bw/day) | 847 | *1.62* | *(2.08)* |
| Mother is multiparous | 1074 | 55.3 | [594] |
| ***Birth factors*** |  |  |  |
| Cesarean birth | 1074 | 31.2 | [335] |
| Length of gestation (weeks) | 1074 | 39.4 | (1.52) |
| Birthweight (kg) | 1072 | 3.53 | (0.52) |

**Supplementary Table 1 | Distribution of key characteristics in the Barwon Infant Study.**

*Note.* SD = standard deviation; GM = geometric mean; GSD = geometric standard deviation; BPA = Bisphenol A; ASD = autism spectrum diagnosis; DEHP = Diethylhexyl phthalate; bw = bodyweight; CBCL = Child Behavior Checklist for Ages 1.5-5.

^1^ Assigned either ‘male’ or ‘female’ at birth based on visible external anatomy

^2^ Above median based on normative data for the CBCL, that is, a T-score above 50 on the DSM-5-oriented autism spectrum problems scale

**^3^** As verified by June 30, 2023

| **Index (unit)[n]** | **Bisphenol A** |
| --- | --- |
| MDL (µg/L) | 0.24 |
| LOR (µg/L) | 0.79 |
| Intra-Day [n=8] |  |
| QCL |  |
| Accuracy | 89 |
| %CV | 6 |
| QCH |  |
| Accuracy | 94 |
| %CV | 7 |
| Inter-Day [n=18] |  |
| QCL |  |
| Accuracy | 99 |
| %CV | 14 |
| QCH |  |
| Accuracy | 95 |
| %CV | 10 |
| SRM |  |
| Accuracy | 88 |
| %CV | 26 |
| Range (µg/L) | 0.1 – 200 |
| Linearity | 0.9979 |
| %[n]>MDL | 53.8 [453] |
| GM (µg/L) | 0.65 |
| 25^th^ percentile (µg/L) | <MDL |
| Median (µg/L) | 0.68 |
| 75^th^ percentile (µg/L) | 2.17 |
| Range of values detected (µg/L) | <MDL, 57.06 |

**Supplementary Table 2 | Quality control information for measurement of BPA and distribution of specific gravity-corrected measurements in urine levels (µg/L) of 842 mothers at 36 weeks’ gestation in the Barwon Infant Study.**

*Note.* MDL = method detection limit; LOR = limit of reporting; QCL = low level quality control; QCH = high level quality control; CV = coefficient of variation; GM = geometric mean.

| **Characteristic** | **N** | **Mean (SD) or % [n]** | |
| --- | --- | --- | --- |
| Child sex: male | 727 | 48.3 | [351] |
| Prenatal BPA | 375 | 3.03 | (4.15) |
| Prenatal BPA: BPA > 4.0 | 375 | 19.7 | [74] |
| Prenatal BPA: BPA < 1.0 | 375 | 22.1 | [83] |
| Age at BPA measurement (gestational weeks) | 371 | 39.3 | (1.27) |
| ***Prenatal factors*** |  |  |  |
| Mother’s age at conception (years) | 727 | 25.2 | (4.94) |
| Mother’s education is high-school and above | 723 | 60.4 | [437] |
| Mother’s ethnicity: Dominican | 727 | 65.1 | [473] |
| Maternal environmental tobacco smoke | 717 | 34.3 | [246] |
| ***Birth factors*** |  |  |  |
| Cesarean birth | 648 | 23.5 | [152] |
| Length of gestation (weeks) | 717 | 39.3 | (1.39) |
| Birthweight (kg) | 670 | 3.37 | (0.48) |

**Supplementary Table 3 | Distribution of key characteristics in the CCCEH-MN cohort**

*Note.* SD = standard deviation; BPA = Bisphenol A;

|  | **Above median CBCL autism spectrum problems^1^** | | | **ASD diagnosis (9 years)** | | |
| --- | --- | --- | --- | --- | --- | --- |
|  | **n** | **matched OR (95% CI)^2^** | ***P*-value** | **n** | **matched OR (95% CI)^3^** | ***P*-value** |
| **BPA (top quartile vs. bottom three quartiles)** |  |  |  |  |  |  |
| Entire sample | 605 | 1.33 (0.91, 1.94) | 0.15 | 306 | 1.04 (0.44, 2.48) | 0.92 |
| Sample stratified by sex^4^ |  |  |  |  |  |  |
| *Boys* | 329 | 1.35 (0.81, 2.26) | 0.25 | 216 | 1.16 (0.42, 3.19) | 0.77 |
| *Girls* | 276 | 1.29 (0.73, 2.29) | 0.38 | 90 | 0.74 (0.14, 3.89) | 0.72 |
| Sample stratified by sex and unweighted aromatase activity score^5^ |  |  |  |  |  |  |
| *Boys, low* | 83 | 3.56 (1.13, 11.22) | 0.03 | 45 | 6.24 (1.02, 38.26) | 0.05 |
| *Boys, high* | 238 | 1.05 (0.58, 1.89) | 0.88 | 171 | 0.59 (0.16, 2.19) | 0.43 |
| *Girls, low* | 72 | 0.95 (0.26, 3.54) | 0.94 | 18 | Too few cases |  |
| *Girls, high* | 199 | 1.40 (0.74, 2.66) | 0.31 | 72 | 1.37 (0.21, 8.90) | 0.74 |
| ***Sensitivity analysis:*** |  |  |  |  |  |  |
| Sample stratified by sex and weighted aromatase activity score^6^ |  |  |  |  |  |  |
| *Boys, low* | 83 | 3.74 (1.12, 12.5) | 0.03 | 54 | 6.06 (0.93, 39.43) | 0.06 |
| *Boys, high* | 238 | 1.04 (0.58, 1.87) | 0.90 | 161 | 0.58 (0.16, 2.09) | 0.40 |
| *Girls, low* | 69 | 1.51 (0.45, 5.08) | 0.50 | 18 | Too few cases |  |
| *Girls, high* | 202 | 1.28 (0.67, 2.46) | 0.45 | 72 | 1.51 (0.24, 9.45) | 0.66 |

**Supplementary Table 4| Conditional logistic regression estimates for association of BPA and ASD outcomes in human whole cohort and subgroup analyses.**

*Note.* CBCL = Child Behavior Checklist for Ages 1.5-5; BPA = Bisphenol A; OR = odds ratio;

^1^Above median based on normative data for the CBCL, that is, a T-score above 50 on the DSM-5-oriented autism spectrum problems scale;

^2^ Participants were matched on assigned sex at birth (in full sample only), ancestry (all four grandparents are Caucasian vs not) and time of day of maternal urine collection (after 2pm vs before);

^3^ Participants were first matched on assigned sex at birth (in full sample only), ancestry (all four grandparents are Caucasian vs not) and time of day of maternal urine collection (after 2pm vs before) and then within these strata, each case was matched to 8 controls based on nearest date and age of interview (see Supplementary Materials and Methods for further details on matching algorithm);

^4^ Assigned either ‘male’ or ‘female’ at birth based on visible external anatomy;

^5^ *CYP19A1* unweighted genetic score for aromatase enzyme activity where ‘low aromatase activity’ means being in the top quartile and ‘high aromatase activity’ means being in the lower three quartiles of an unweighted sum of the following genotypes associated with lower estrogen levels^28^ (participant given 1 if genotype is present, 0 if not): CC of rs12148604, GG of rs4441215, CC of rs11632903, CC of rs752760, AA of rs2445768;

^6^ *CYP19A1* weighted genetic score for aromatase gene expression where ‘low aromatase activity’ means being in the top quartile and ‘high aromatase activity’ means being in the lower three quartiles of the sum of the following genotypes associated with *CYP19A1* expression, weighted by their normalized effect size in amygdala tissue (weighting multiplied by number of effect alleles, 0, 1, or 2, for each variant for the participant) and multiplied by -1 so that higher values indicate lower aromatase activity: AA of rs7169770, CC of rs1065778, AA of rs28757202, CC of rs12917091, AA of rs3784307.

| **Sample** | **Exposure-outcome association** | **Potential confounders tested individually** | **Sample size** | **Odds ratio (95% CI)** | ***P*-value** | **% change in exposure-outcome association (log odds) after addition of potential confounder^1^** |
| --- | --- | --- | --- | --- | --- | --- |
| Males with low aromatase enzyme activity | Prenatal BPA exposure and CBCL autism spectrum problems at 2 years | Household income under $100K | 80 | 3.61 (1.12, 11.68) | 0.03 | -4.93 |
|  |  | Mother’s age at conception | 83 | 3.58 (1.13, 11.30) | 0.03 | 0.36 |
|  |  | Mother is university-educated | 82 | 3.13 (0.97, 10.17) | 0.06 | -9.09 |
|  |  | Father is university-educated | 82 | 3.45 (1.09, 10.94) | 0.04 | -1.80 |
|  |  | Prenatal maternal smoking | 83 | 3.57 (1.13, 11.25) | 0.03 | 0.07 |
|  |  | Prenatal maternal alcohol use | 80 | 3.73 (1.16, 12.01) | 0.03 | 0.32 |
|  |  | Prenatal DEHP exposure | 83 | 3.85 (1.16, 12.80) | 0.03 | 6.14 |
|  |  | Mother is multiparous | 83 | 3.53 (1.12, 11.12) | 0.03 | -0.79 |
|  |  | Caesarean birth | 83 | 3.56 (1.13, 11.24) | 0.03 | -0.12 |
|  |  | Length of gestation | 83 | 3.44 (1.07, 11.06) | 0.04 | -2.65 |
|  |  | Birthweight | 83 | 3.59 (1.10, 11.70) | 0.03 | 0.52 |

**Supplementary Table 5 | Sensitivity analysis of additional potential sources of confounding.** Given the comprehensive data collected on participants in the Barwon Infant Study, a sensitivity analysis was conducted to test the robustness of the findings to other potential sources of confounding (not already controlled for in the original matched analysis where we matched on ancestry and time of day of maternal urine collection). We used the change-in-estimate approach^29, 30^. Addition of further individual potential confounders to the model did not materially alter the matched odds ratio for the BPA and CBCL autism spectrum problems association in males with low aromatase enzyme activity. This sensitivity analysis was conducted for ASD symptoms at age 2 as the outcome where the sample size was sufficient, that is over 80 participants after the addition of the potential confounder.

*Note.* DEHP = Diethylhexyl phthalate.

^1^ Change in estimate calculated comparing models with and without the potential confounder using the same sample, that is, participants with complete data for the original variables in the model and the potential confounder.

**Supplementary References**

1. Pidsley R*, et al.* Critical evaluation of the Illumina MethylationEPIC BeadChip microarray for whole-genome DNA methylation profiling. *Genome biology* **17**, 208 (2016).

2. Phipson B, Maksimovic J, Oshlack A. missMethyl: an R package for analyzing data from Illumina's HumanMethylation450 platform. *Bioinformatics (Oxford, England)* **32**, 286-288 (2016).

3. Aryee MJ*, et al.* Minfi: a flexible and comprehensive Bioconductor package for the analysis of Infinium DNA methylation microarrays. *Bioinformatics (Oxford, England)* **30**, 1363-1369 (2014).

4. Gentleman RC*, et al.* Bioconductor: open software development for computational biology and bioinformatics. *Genome biology* **5**, R80 (2004).

5. Maksimovic J, Gordon L, Oshlack A. SWAN: Subset-quantile Within Array Normalization for Illumina Infinium HumanMethylation450 BeadChips. *Genome biology* **13**, R44 (2012).

6. Bakulski KM*, et al.* DNA methylation of cord blood cell types: applications for mixed cell birth studies. *Epigenetics* **11**, 354-362 (2016).

7. Morin AM*, et al.* Maternal blood contamination of collected cord blood can be identified using DNA methylation at three CpGs. *Clin Epigenetics* **9**, 75 (2017).

8. Anthoni H*, et al.* The aromatase gene CYP19A1: several genetic and functional lines of evidence supporting a role in reading, speech and language. *Behav Genet* **42**, 509-527 (2012).

9. Information NCfB. CYP19A1 cytochrome P450 family 19 subfamily A member 1 [ Homo sapiens (human) ].) (2021).

10. Sheng X*, et al.* Systematic integrated analysis of genetic and epigenetic variation in diabetic kidney disease. *Proc Natl Acad Sci U S A* **117**, 29013-29024 (2020).

11. Harada N, Honda S-i. Analysis of spatiotemporal regulation of aromatase in the brain using transgenic mice. *J Steroid Biochem Mol Biol* **95**, 49-55 (2005).

12. Byers SL, Wiles MV, Dunn SL, Taft RA. Mouse estrous cycle identification tool and images. *PLoS One* **7**, e35538 (2012).

13. Paxinos G, Franklin KBJ. *Paxinos and Franklin's the Mouse Brain in Stereotaxic Coordinates*. Elsevier Science (2019).

14. Schmitz C, Hof PR. Design-based stereology in neuroscience. *Neuroscience* **130**, 813-831 (2005).

15. Namba T, Nardelli J, Gressens P, Huttner WB. Metabolic regulation of neocortical expansion in development and evolution. *Neuron* **109**, 408-419 (2021).

16. Frye RE*, et al.* Prenatal air pollution influences neurodevelopment and behavior in autism spectrum disorder by modulating mitochondrial physiology. *Mol Psychiatry* **26**, 1561-1577 (2021).

17. DeRosa BA*, et al.* Convergent pathways in idiopathic autism revealed by time course transcriptomic analysis of patient-derived neurons. *Sci Rep* **8**, 8423 (2018).

18. Sarachana T, Hu VW. Genome-wide identification of transcriptional targets of RORA reveals direct regulation of multiple genes associated with autism spectrum disorder. *Mol Autism* **4**, 14 (2013).

19. Schumann CM, Amaral DG. Stereological analysis of amygdala neuron number in autism. *J Neurosci* **26**, 7674-7679 (2006).

20. Avino TA*, et al.* Neuron numbers increase in the human amygdala from birth to adulthood, but not in autism. *Proc Natl Acad Sci U S A* **115**, 3710-3715 (2018).

21. Herrington JD, Miller JS, Pandey J, Schultz RT. Anxiety and social deficits have distinct relationships with amygdala function in autism spectrum disorder. *Soc Cogn Affect Neurosci* **11**, 907-914 (2016).

22. Stoner R*, et al.* Patches of disorganization in the neocortex of children with autism. *N Engl J Med* **370**, 1209-1219 (2014).

23. Wang J, Barstein J, Ethridge LE, Mosconi MW, Takarae Y, Sweeney JA. Resting state EEG abnormalities in autism spectrum disorders. *J Neurodev Disord* **5**, 24 (2013).

24. Alia-Klein N*, et al.* Human cognitive ability is modulated by aromatase availability in the brain in a sex-specific manner. *Front Neurosci* **14**, 565668 (2020).

25. Schumann CM, Amaral DG. Stereological analysis of amygdala neuron number in autism. *J Neurosci* **26**, 7674-7679 (2006).

26. Brocca ME, Pietranera L, Roig P, Lima A, De Nicola AF. Effects of 17β-estradiol on the cytoarchitecture of pyramidal CA1 neurons in normoglycemic and diabetic male spontaneously hypertensive rats. *Neuroscience* **280**, 243-253 (2014).

27. Matuszczak E, Komarowska MD, Debek W, Hermanowicz A. The Impact of Bisphenol A on Fertility, Reproductive System, and Development: A Review of the Literature. *International journal of endocrinology* **2019**, 4068717 (2019).

28. Kidokoro K*, et al.* Association between CYP19A1 polymorphisms and sex hormones in postmenopausal Japanese women. *J Hum Genet* **54**, 78-85 (2009).

29. Lash TL, VanderWeele TJ, Haneuse S, Rothman KJ. Modern Epidemiology.). 4th_Edition edn. Lippincott Williams & Wilkins (2021).

30. Greenland S, Pearce N. Statistical foundations for model-based adjustments. *Annu Rev Public Health* **36**, 89-108 (2015).
