# Supplementary material for: Male autism spectrum disorder is linked to brain aromatase disruption by prenatal BPA in multimodal investigations and 10HDA ameliorates the related mouse phenotype
Source: Nat Commun. 2024 Aug 7;15:6367. doi: 10.1038/s41467-024-48897-8 (PMC11306638; doi:10.1038/s41467-024-48897-8)
Supplement: Supplementary file 3 — Description of Additional Supplementary Files [file 41467_2024_48897_MOESM3_ESM.pdf]

Description of Additional Supplementary Files

Supplementary Dataset 1: Tab 1: Differentially Expressed Genes in cortical primary cell culture from BPA exposed mouse embryos. male BPA exposed vs male control. Tab 2: RNASeq list of Differentially Expressed Genes in cortical primary cell culture from untreated male mouse embryos, cells treated with 10HDA. Male 10HDA vs male control

Supplementary Dataset 2: List of oligonucleotides used for genotyping

Supplementary Movies

Supplementary Movie 1: Video demonstrating Molecular docking of estrogen receptor β with E2 superimposed with BPA.

Supplementary Movie 2: Video demonstrating Molecular docking of estrogen receptor β with E2 superimposed with 10HDA.
