# Supplementary material for: Male autism spectrum disorder is linked to brain aromatase disruption by prenatal BPA in multimodal investigations and 10HDA ameliorates the related mouse phenotype
Source: Nat Commun. 2024 Aug 7;15:6367. doi: 10.1038/s41467-024-48897-8 (PMC11306638; doi:10.1038/s41467-024-48897-8)
Supplement: Supplementary file 8 — Reporting Summary [file 41467_2024_48897_MOESM8_ESM.pdf]

nature portfolio

Corresponding author(s): Anne-Louise Ponsonby and Wah Chin Boon

Last updated by author(s): Mar 15, 2024

Reporting Summary

Nature Portfolio wishes to improve the reproducibility of the work that we publish. This form provides structure for consistency and transparency in reporting. For further information on Nature Portfolio policies, see our Editorial Policies and the Editorial Policy Checklist.

Statistics

For all statistical analyses, confirm that the following items are present in the figure legend, table legend, main text, or Methods section.

n/a	Confirmed	
☐	☒	The exact sample size (*n*) for each experimental group/condition, given as a discrete number and unit of measurement
☐	☒	A statement on whether measurements were taken from distinct samples or whether the same sample was measured repeatedly
☐	☒	The statistical test(s) used AND whether they are one- or two-sided<br>*Only common tests should be described solely by name; describe more complex techniques in the Methods section.*
☐	☒	A description of all covariates tested
☐	☒	A description of any assumptions or corrections, such as tests of normality and adjustment for multiple comparisons
☐	☒	A full description of the statistical parameters including central tendency (e.g. means) or other basic estimates (e.g. regression coefficient) AND variation (e.g. standard deviation) or associated estimates of uncertainty (e.g. confidence intervals)
☐	☒	For null hypothesis testing, the test statistic (e.g. *F*, *t*, *r*) with confidence intervals, effect sizes, degrees of freedom and *P* value noted<br>*Give P values as exact values whenever suitable.*
☒	☐	For Bayesian analysis, information on the choice of priors and Markov chain Monte Carlo settings
☒	☐	For hierarchical and complex designs, identification of the appropriate level for tests and full reporting of outcomes
☒	☐	Estimates of effect sizes (e.g. Cohen's *d*, Pearson's *r*), indicating how they were calculated

Our web collection on statistics for biologists contains articles on many of the points above.

Software and code

Policy information about availability of computer code

Data collection	TopScan 1.0, Neuroexplorer, Neurolucida, SteroinvestigatorTM 8.5, LTP-Director, LTP-Analyzer, Sirenia Pro, DockThor, Odyssey infrared imaging system, Image Studio Lite, Pinnacle EEG/EMG tethered recording system, LTP-Director, LTP-Director
Data analysis	GraphPad Prism 9.4 was used for graphing data and statistical analysis of in vitro and in vivo studies, except fR was used for graphing data and statistical analysis where GEE was used in R v4.1.2. . Ingenuity was used for pathway analysis, clusterProfileR in R v3.6.3 for additional pathway analysis. Human data was analysed using Stata 15.1 and R v3.6.3.

For manuscripts utilizing custom algorithms or software that are central to the research but not yet described in published literature, software must be made available to editors and reviewers. We strongly encourage code deposition in a community repository (e.g. GitHub). See the Nature Portfolio guidelines for submitting code & software for further information.

Data

Policy information about availability of data

All manuscripts must include a data availability statement. This statement should provide the following information, where applicable:

- Accession codes, unique identifiers, or web links for publicly available datasets
- A description of any restrictions on data availability
- For clinical datasets or third party data, please ensure that the statement adheres to our policy

For laboratory data access contact the corresponding author. Access to BIS data, including all data used in this paper, can be requested through the BIS Steering

Committee by contacting annelouise.ponsonby@florey.edu.au. Requests to access cohort data are considered on scientific and ethical grounds and, if approved, provided under collaborative research agreements. Additional project information, including cohort data description and access procedure, is available at the project's website https://www.barwoninfantstudy.org.au.

Research involving human participants, their data, or biological material

Policy information about studies with human participants or human data. See also policy information about sex, gender (identity/presentation), and sexual orientation and race, ethnicity and racism.

Reporting on sex and gender	Sex has been defined in the methods and results following the current Nature guidelines. We have stated how sex was determined and we have stated how sex was assigned in both the methods and the tables footnotes reporting sex. Yes, sex-based analysis have been reported. No gender-based analysis were conducted and gender is not referred to in this paper.
Reporting on race, ethnicity, or other socially relevant groupings	We have used direct sociodemographic variables. We do not use race or ethnicity as a proxy for other socially constructed variables. Clear definitions of the relevant terms are provided. These include self reported ancestry in table S3. We also matched on other self-reported characteristics such as maternal age in the replication cohort and the key 2015 PNAS(ref) publication that we defined ethnicity as referring to self-reported ancestry e.g. In the BIS cohort, ethnicity was defined as all four grandparents are Caucasian vs not (see Table S3). For the CCCEH-MN cohort, ethnicity was defined as Dominican vs African American.
Population characteristics	Population covariates are provided in Supplementary Table 1 and Supplementary Table 2, and referred to in the abstract and results sections.
Recruitment	Barwon Infant Study: Participants were recruited from two hospitals (Geelong Hospital and St John of God Hospital) in the Barwon region from which more than 90% of the live births occur. Pregnant women attending their antenatal book in appointment at approximately 15 weeks were invited to participate in BIS.<br><br>Columbia Center for Children's Environmental Health: Women were recruited from prenatal ambulatory care clinics of New York Presbyterian Medical Center or Harlem Hospital. The urban neighborhoods of Washington Heights, Central Harlem and the South Bronx in New York City are the catchment areas for these two clinics.
Ethics oversight	The human Barwon Infant Study cohort study was approved by the Barwon Health Human Research Ethics Committee, and families provided written informed consent. Parents or guardians provided written informed consent at prenatal recruitment and again when the child was 2 years of age. The human Columbia Center for Children's Environmental Health Mothers and Newborn cohort study was approved by the Institutional Review Boards of Columbia University and the Centers for Disease Control and Prevention, and all participants in the study provided informed consent.

Note that full information on the approval of the study protocol must also be provided in the manuscript.

Field-specific reporting

Please select the one below that is the best fit for your research. If you are not sure, read the appropriate sections before making your selection.

☒ Life sciences ☐ Behavioural & social sciences ☐ Ecological, evolutionary & environmental sciences

For a reference copy of the document with all sections, see nature.com/documents/nr-reporting-summary-flat.pdf

Life sciences study design

All studies must disclose on these points even when the disclosure is negative.

Sample size	Human cohorts work: The BIS cohort was originally designed to investigate multiple outcomes. It was designed to detect a risk ratio of 2.2 or more for ADHD and 2.7 or more for ASD with 80% power for the top chemical quartile. The CCCEH study utilized EWAS samples, out of the original 727 samples, 209 samples had DNA methylation data and covariate data.<br><br>Experimental laboratory work: Sample sizes were chosen based on power analysis on data from preliminary experiments, and prior literature and experience (ref: Sample et al, Behav Brain Res. 2017, https://linkinghub.elsevier.com/retrieve/pii/S0166-4328(16)30647-7).<br>based on prior literature and experience. For behavioural experiments, n=6-10/group. For behavioural experiments involving prenatal BPA exposure, the sample size was determined by the number of treated dams, rather than the number of offspring (reference: Wolstenholme, et al., PLoS One, 2011. https://www.ncbi.nlm.nih.gov/pmc/articles/PMC3182223/). At least 3 independent replicates were done for cell and histology work.
Data exclusions	Human cohorts work: The BIS cohort excluded infants born under 32 weeks gestation or infants with a major congenital or clinical abnormality<br>Experimental laboratory work: We excluded animals which developed a disease or showed general clinical signs.
Replication	Human cohorts work: The BIS epigenetic findings were replicated in the CCCEH study. Biological measures were derived using independent biological replicates.<br>Experimental laboratory work: All experimental results were derived by using independent biological replicates. BPA and ArKO experiments was replicated independently - two independent cohorts during different times by two different investigators (ArKO = Kris Vacy and Chrissy H. BPA = Kris Vacy and Jessalyn)

Randomization	Human cohorts work: Human birth cohorts were prospective and observational in design and therefore they are non randomised for prenatal BPA exposure.<br>Experimental laboratory work: In the BPA treatment experiments, mice were randomly assigned to vehicle or BPA exposure, matched by weight gain at E9.5 and litter/cage where applicable. In the 10-HDA treatment experiments, mice were randomly assigned to vehicle or 10HDA exposure, matched on sex and litter. In the E2 treatment experiments, ArKO mice were implanted with sham or E2 pallet prior to genotyping and could not be randomly assigned.
Blinding	Human cohorts work: Child outcome assessment was blind to prenatal BPA exposure levels.<br>Experimental laboratory work: Treatment was administered unblinded investigators. Investigators were blind to treatment - did not know the treatment or genotype status of the animal, group during the conduct of the experiment and assessment of the outcome.

Reporting for specific materials, systems and methods

We require information from authors about some types of materials, experimental systems and methods used in many studies. Here, indicate whether each material, system or method listed is relevant to your study. If you are not sure if a list item applies to your research, read the appropriate section before selecting a response.

Materials & experimental systems

n/a	Involved in the study
☐	☒ Antibodies
☐	☒ Eukaryotic cell lines
☒	☐ Palaeontology and archaeology
☐	☒ Animals and other organisms
☒	☐ Clinical data
☒	☐ Dual use research of concern
☒	☐ Plants

Methods

n/a	Involved in the study
☒	☐ ChIP-seq
☒	☐ Flow cytometry
☒	☐ MRI-based neuroimaging

Antibodies

Antibodies used	Primary antibody rabbit Anti-Aromatase (1:2000; cat# A7981; Sigma Aldrich, St. Louis, MO, USA).<br>Secondary antibody Goat anti-Rabbit IRDye 800CW (cat# 926-32211; LI-COR Biosciences, Lincoln, NE, USA.)<br>Primary antibody mouse Anti-β-Actin (1:10,000; cat# A5316; Sigma Aldrich, St. Louis, MO, USA).<br>Secondary antibody Goat anti-Mouse IRDye 680RD (cat# 926-68070; LI-COR, Biosciences, Lincoln, NE, USA)<br>Primary antibody chicken anti-EGFP (1:1,000; cat# ab13970; Abcam, United Kingdom)<br>Primary antibody rabbit anti-c-Fos (1:2000; cat# ABE457; Calbiochem, USA)<br>Secondary antibody donkey anti-chicken Alexa488 (1:200; cat# A78948, Invitrogen, USA)<br>Secondary antibody donkey anti-rabbit Alexa594 (1:200; cat# A-21207; Invitrogen, USA)<br>Primary antibody anti-βIII tubulin monoclonal (1:1000; cat #ab41489, Abcam, United Kingdom)<br>Secondary antibody goat anti-mouse Alexa Fluor 488, ((1:2000; cat#A11017; Invitrogen, USA)
Validation	Antibodies were commercially validated. See links:<br>Primary antibody Anti-Aromatase: https://www.sigmaaldrich.com/AU/en/product/sigma/a7981<br>Secondary antibody IRDye 800CW: https://www.licor.com/bio/reagents/irdye-800cw-goat-anti-rabbit-igg-secondary-antibody<br>rimary antibody Anti-β-Actin: https://www.sigmaaldrich.com/AU/en/product/sigma/a5316<br>secondary antibody IRDye 680RD: https://www.licor.com/bio/reagents/irdye-680rd-goat-anti-mouse-igg-secondary-antibody<br>chicken anti-EGFP primary antibody: https://www.abcam.com/en-au/products/primary-antibodies/gfp-antibody-ab13970#application=wb<br>Primary antibody rabbit anti-c-Fos: https://www.merckmillipore.com/AU/en/product/Anti-c-Fos-Antibody,MM_NF-ABE457<br>Secondary antibody donkey anti-chicken Alexa488: https://www.thermofisher.com/antibody/product/Donkey-anti-Chicken-IgY-H-L-Highly-Cross-Adsorbed-Secondary-Antibody-Polyclonal/A78948<br>Secondary antibody donkey anti-rabbit Alexa594: https://www.thermofisher.com/antibody/product/Donkey-anti-Rabbit-IgG-H-L-Highly-Cross-Adsorbed-Secondary-Antibody-Polyclonal/A-21207<br>Primary antibody anti-βIII tubulin monoclonal: https://www.abcam.com/en-au/products/primary-antibodies/beta-iii-tubulin-antibody-ab41489#<br>Secondary antibody goat anti-mouse Alexa Fluor 488: https://www.thermofisher.com/antibody/product/Goat-anti-Mouse-IgG-H-L-Cross-Adsorbed-Secondary-Antibody-Polyclonal/A-11017

Eukaryotic cell lines

Policy information about cell lines and Sex and Gender in Research

Cell line source(s)	The female human neuroblastoma line SH-SY5Y was used in the western blotting experiment - source: CRL-2266, American

Cell line source(s)	Type Culture Collection, Virginia, USA.<br>Primary cortical cell lines were taken from male mice at ED15.5.
Authentication	Cell lines were not authenticated
Mycoplasma contamination	All cell lines tested negative for mycoplasma
Commonly misidentified lines<br>(See ICLAC register)	No commonly misidentified lines were used in this study

Animals and other research organisms

Policy information about studies involving animals; ARRIVE guidelines recommended for reporting animal research, and Sex and Gender in Research

Laboratory animals	21 week old to 8 month old C57Bl/6J and FVB/N mice were used in this study. ArKO mice were bred on a C57Bl/6J background, and Cyp19 EGFP reporter mice on an FVB/N background. Mice were maintained under a 12-hours light/12-hours dar cycle at a constant temp (21°C) with free access to food and water. Mice were kept on a soyfree diet to reduce exposure to phytoestrogens.
Wild animals	This study did not involve wild animals.
Reporting on sex	Both sexes were analyzed as separate groups. Some experiments only involved male animals due to further testing of the phenotype present in male mice.
Field-collected samples	This study did not involve animal or other research organism samples collected from the field.
Ethics oversight	All animal studies were approved by the Florey Animal Ethics Committee.

Note that full information on the approval of the study protocol must also be provided in the manuscript.
